# Psychological Symptom Progression in School-Aged Children After COVID-19 Home Confinement: A Longitudinal Study

**DOI:** 10.3389/fpsyt.2022.809107

**Published:** 2022-04-13

**Authors:** Xinyan Xie, Qi Liu, Kaiheng Zhu, Qi Jiang, Yanan Feng, Pei Xiao, Xiaoqian Wu, Ranran Song

**Affiliations:** Department of Maternal and Child Health and Ministry of Education (MOE) Key Lab of Environment and Health, School of Public Health, Tongji Medical College, Huazhong University of Science and Technology, Wuhan, China

**Keywords:** depressive symptom, anxiety symptom, school-aged children, coronavirus disease 2019, longitudinal study

## Abstract

**Background:**

The long-term mental health effects of coronavirus disease 2019 (COVID-19) in children are rarely reported. We aimed to investigate the progression of depressive and anxiety symptoms among a cohort of children in the initial epicenter of COVID-19 in China.

**Methods:**

Two waves of surveys were conducted in the same two primary schools in Wuhan and Huangshi, Hubei province: Wave 1 from 28 February to 5 March, 2020 (children had been confined to home for 30–40 days) and Wave 2 from 27 November to 9 December, 2020 (schools had reopened for nearly 3 months). Depressive and anxiety symptoms were estimated using the Children's Depression Inventory – Short Form (CDI-S) and the Screen for Child Anxiety Related Emotional Disorders (SCARED), respectively. ΔCDI-S and ΔSCARED scores between Wave 2 and Wave 1 were calculated and further categorized into tertiles. Multivariable linear regression and multinomial logistic regression models were then applied.

**Results:**

A total of 1,224 children completed both surveys. The prevalence of mental health outcomes at Wave 2 increased significantly compared to Wave 1, specifically depressive symptoms (age-standardized prevalence rates: 37.5 vs. 21.8%) and anxiety symptoms (age-standardized prevalence rates: 24.0 vs. 19.6%). Higher ΔSCARED scores were observed in females and children in Wuhan, and children with experience of neglect had higher ΔCDI-S (β = 1.12; 95% CI = 0.67–1.58) and ΔSCARED (β = 6.46; 95% CI = 4.73–8.19) scores compared with those without experience of neglect. When the Δ scores were further categorized into tertiles, similar results were found.

**Conclusions:**

The prevalence of depressive and anxiety symptoms after schools resumed was increased compared with that during the home quarantine period, even though the COVID-19 pandemic was under control. Females and children in Wuhan, and also children with experience of neglect were at increased risk of mental health disorders.

## Introduction

Mental health has been increasingly seen as a major public health problem. It is estimated that between 10 and 20% of children and adolescents suffer from some type of mental health disorder (1). As most mental health disorders begin in childhood, a sensitive period of child development, early identification and treatment of mental health needs during this time is essential (2).

The coronavirus disease 2019 (COVID-19) outbreak was declared a pandemic by the World Health Organization (WHO), and COVID-19 emergency measures (i.e., city-wide lockdown) began in Wuhan, Hubei province on 23 January, 2020. This was eventually followed by other cities in Hubei province (3, 4). According to the Ministry of Education, the COVID-19 pandemic has caused long-term home restrictions for 180 million primary and secondary school students (5). In Hubei province, primary schools have been closed and shifted to home-based distance-learning models for the whole Spring semester. Hence, children did not have face-to-face learning until September 2020. Recent literature suggested that COVID-19 itself, along with school closures and home quarantine caused by COVID-19, has adversely affected children's mental health (6–9). COVID-19 has become a major global threat, impacting the mental well-being of children (10, 11). A series of studies from Effects of home Confinement on multiple Lifestyle Behaviours during the COVID-19 outbreak (ECLB-COVID19), an international online survey on mental health and multi-dimensional lifestyle behaviors during home confinement, have also highlighted the significant impact that home confinement has had on health, mental well-being, mood, life satisfaction, and multidimensional lifestyle behaviors (12–17). COVID-19 home confinement has negatively impacted mental health, with a greater proportion of people experiencing psychosocial and emotional disorders (14).

A range of mental health problems have accompanied the pandemic, such as depressive/anxiety disorders and post-traumatic stress disorder (PTSD) (18). For instance, isolated children had average PTSD scores that were four times higher than those of children who were not isolated (19). The mental health problems of children could continue into adulthood and adversely affect their physical and mental health (11). Depressive and anxiety symptoms are considered to be the early stages of major depressive disorder and anxiety disorder (20, 21), both of which could lead to poor academic performance, impaired cognitive function, social problems, and impaired psychosocial functions (20–22). The COVID-19 pandemic and the related measures against it, including self-isolation, quarantine, and social distancing, could have a detrimental impact on mental health. Individuals had to face significant changes in everyday life, possibly causing acute fight-or-flight responses (23). Uncertainty, fear, and discrimination toward infected people and their family members might generate psychological consequences that would need to be addressed by professionals and psychiatrists (24). The psychiatric problems that accompanied COVID-19 might therefore be a marathon rather than a sprint (25).

Until now, the majority of existing studies have focused on cross-sectional data, which cannot examine the long-term impact of COVID-19 over time (26–28). Our previous cross-sectional study conducted between 28 February and 5 March 2020 found that the prevalence of depressive (17.2%) and anxiety (18.9%) symptoms of children in Hubei province was higher than from other surveys in China (6). One longitudinal cohort study of children and adolescents in an area of China with a low risk of COVID-19 showed that the prevalence of psychological symptoms was higher after school reopening (on May 2020) than before the COVID-19 outbreak (29). Therefore, there is an urgent need for long-term follow-up studies on the psychological symptoms of school-aged children, especially those in the high risk area of the COVID-19 outbreak (30). We aimed to examine depressive and anxiety symptoms among a cohort of children after school reopening in Wuhan and Huangshi, Hubei province, China based on our previous study about the mental health status of children during the COVID-19 outbreak (6). We hypothesized that the impact of COVID-19 on the mental health of children may be long term and that the mental health status of children may worsen over time.

## Materials and Methods

### Study Design

At Wave 1, we conducted the survey among children in Grades 2–6 at two primary schools in Hubei province from 28 February to 5 March 2020 through an online crowd-sourcing platform. At that time, children had been confined in their home for 30–40 days. Children took the online survey after their guardian agreed to the statement “I permit my child to participate in the survey” in the survey link. Detailed information were shown in our previous article (6).

At Wave 2, we conducted the second survey at the same schools between 27 November and 9 December 2020 on site. At that time, cities had been unsealed for nearly 7 months and schools had reopened for nearly 3 months. We obtained oral informed consent from parents by inquiring through head teachers. The investigators organized children to independently accomplish the questionnaires in class and encouraged them to complete the questionnaire as much as possible.

This study was approved by the Ethics Committee of Tongji Medical College, Huazhong University of Science and Technology. Informed consent of the children and their guardians was obtained after the nature of the procedures had been fully explained. There was no disclosed information that might identify a particular person. All procedures performed in studies involving human participants were in accordance with the 1964 Helsinki declaration and its later amendments.

### Study Population

#### Wave 1

A total of 2,330 children in Grades 2–6 from two primary schools in Hubei province were invited to participate the survey and 1,784 participants completed the survey (675 children residing in Wuhan and 1,109 in Huangshi). The response rate was 76.6%. All questionnaires passed the quality audit, and the effective rate was 100.0%.

#### Wave 2

Children were promoted to the next grade in September each year, and all the children in the two primary schools were promoted at Wave 2. Therefore, the second survey started with children in Grade 3. As children in Grade 6 at Wave 1 were promoted from the primary school to the junior middle school, they were not included in the follow-up at Wave 2. A total of 2,245 children in Grades 3–6 from the same schools, including 698 from Wuhan and 1,547 from Huangshi, were invited to participate in the survey at Wave 2. Among these children, 2,211 completed the survey, with a response rate of 98.5%. After a quality audit, 2,209 questionnaires were further analyzed, with an effective rate of 99.9%.

Using student names and IDs, we matched the questionnaires from both waves. There was a total of 1,224 children who completed both surveys, with 689 (56.3%) male and 805 (65.8%) participats who resided in Huangshi. The data from those 1,224 children were used in all analyses.

### Measures

In both surveys, the gender, grade, location of school, and depressive and anxiety symptoms of participants were collected. In China, children aged 6 enter primary school and are about 11 years old when they are in grade 6. Thus, the grade could be a good approximation of age. Detailed information was shown in our previous article (6). Depressive and anxiety symptoms were measured using the Children's Depression Inventory—Short Form (CDI-S) and the Screen for Child Anxiety Related Emotional Disorders (SCARED), respectively. Additionally, at Wave 1, COVID-19-related questions were collected. At Wave 2, the daily sleep time in the past week and experience of neglect in the previous year were measured and collected via five items in the Conflict Tactics Scales, Parent-child Version (CTSPC).

#### Depressive symptoms

Depressive symptoms were estimated using the CDI-S at Waves 1 and 2 (31). The CDI-S consists of 10 items, each with a score of 0–2. Each item requires respondents to rate the severity of each symptom of depression. The CDI-S has shown good internal consistency (Cronbach's α = 0.75) in the study with Chinese children (32). The total score ranges from 0 to 20. A higher score indicates more severe depressive symptoms, while a CDI-S value of ≥ 4 is defined as depressive symptoms (33). The difference (Δ) in CDI-S score between Wave 2 and Wave 1 was calculated via subtraction, with a positive/negative change representing an increase/decrease of CDI-S score at Wave 2, respectively. Based on the tertiles of the ΔCDI-S score, it is further categorized into low (< 1), moderate (≥ 1, < 3), and high (≥ 3) change.

#### Anxiety Symptoms

Screen for Child Anxiety Related Emotional Disorders is a 41-item self-report instrument that was used to measure anxiety symptoms at Waves 1 and 2 (34). The questionnaire proved to have adequate reliability (retest reliability: 0.567–0.608; internal consistency: 0.890) and fair validity (correlation coefficients from 0.300 to 0.444) (35). Children rate each symptom on a three-point Likert scale: 0 (almost never), 1 (sometimes), and 2 (often). Total scores ranged from 0 to 82, and the accepted cut-off score for anxiety disorder is 23 (35). Children with higher scores have more severe symptoms of anxiety. The ΔSCARED score between Wave 2 and Wave 1 was calculated and was used to indicate an increase/decrease of SCARED score in Wave 2. Based on the tertiles of the ΔSCARED score, it is further categorized into low (< 0), moderate (≥ 0, < 11), and high (≥ 11) change.

#### Neglect

Five items covering neglect behaviors in the CTSPC were used to measure the experience of neglect (36). Children were asked to report their experience of neglect in the preceding year at Wave 2. Thus, children's experience of neglect at the time of the first survey was also covered. The affirmative responses to any item were used to represent self-reported exposure to neglect.

With regard to COVID-19, children were asked to answer two questions at Wave 1: 1) “Which are more likely the host of SARS-CoV-2?,” with choices that include “wild animals,” “domesticated animals,” and “do not know,” and 2) “Which of the following protective measures have you taken during the COVID-19 outbreak?,” with choices that include “Reminding my family members to wear masks,” “Convince my family members not to go out or gathering,” “Ventilating the house frequently,” and “Washing hands frequently.” Children who chose wild animals and those who had taken all protective measures were deemed to know the host of SARS-CoV-2 and how to take protective measures during the COVID-19 pandemic.

### Statistical Analysis

All analyses were performed in Statistical Package for the Social Sciences (SPSS) 22.0 and Microsoft Excel (2016). Both R (v3.2.5) and Microsoft Excel (2016) were used to generate the figures. Frequencies and percentages were summarized for categorical variables. Means and standard deviations were used to describe continuous variables. Age-standardized prevalence rates of depressive and anxiety symptoms were calculated based on the Chinese population from the 2020 China census data (37). We used McNemar's test to evaluate the trend in the prevalence of psychological symptoms between the two waves. We also performed multivariable linear regression models to examine the ΔCDI-S and ΔSCARED scores. Multinomial logistic regression models were applied to examine the tertiles of the ΔCDI-S and ΔSCARED scores. Multiple imputation with 20 times interpolation was carried out for independent variables that had a few nonresponses [daily sleep time (missing data, 20.5%) and neglect behaviors (missing data, 0.8%)]. Sensitivity analysis using the complete data was also performed to evaluate the validity of multiple imputation. The odds ratio (OR), β value, and 95% confidence interval (95% CI) were reported and *p*-values were two-tailed, with a significance level at 0.05.

## Results

Among 1,224 children who completed both surveys, 689 (56.3%) children were males and 805 (65.8%) resided in Huangshi. The average ages of children were 9.32 ± 1.10 years at Wave 1 and 10.07 ± 1.10 years at Wave 2, with 1.1% of children lacking age information. The percentages of participants in Grades 3–6 were 27.5% (337), 23.9% (292), 27.8% (340), and 20.8% (255), respectively. There were 45.8% of children who took all required protective measures during COVID-19 and 70.9% who knew the host of SARS-CoV-2 at Wave 1. Additionally, 54.2% of children had more than 8 h of daily sleep time and 69.8% showed that they had experience of neglect in the preceding year at Wave 2 (Table 1).

**Table 1:** Demographic characteristics of children who completed both surveys.

**Characteristic**	***n***	**Percentage (%)**
Overall	1,224	100.0
**Gender**		
Male	689	56.3
Female	535	43.7
**School location**		
Wuhan	419	34.2
Huangshi	805	65.8
**Grade**		
Grade 3	337	27.5
Grade 4	292	23.9
Grade 5	340	27.8
Grade 6	255	20.8
**Taking all protective measures during COVID-19^a^**	
No	663	54.2
Yes	561	45.8
**Knowing the host of SARS-CoV-2^a^**		
No	356	29.1
Yes	868	70.9
**Daily sleep time^b^**		
<8 h	309	25.2
≥8 h	664	54.2
Missing data	251	20.5
**Parent-Child Tactics Scale Neglect-neglect behaviors^b^**		
Neglect	854	69.8
Non-neglect	360	29.4
Missing data	10	0.8

a *The items were investigated at Wave 1*.

b *The items were investigated at Wave 2*.

Age-standardized prevalence rates of depressive symptoms at Wave 1 and Wave 2 were 21.8 and 37.5%, respectively, and were 19.6 and 24.0%, respectively, for anxiety symptoms. A total of 20.4% (250) of participants had depressive symptoms at Wave 1 and 39.8% (487) at Wave 2. The average score of the CDI-S rose from 2.22 (2.49) for Wave 1 to 3.57 (3.29) for Wave 2. For the anxiety symptoms, 19% (232) of children were detected at Wave 1 and 33.2% (406) were detected at Wave 2. The average score of SCARED were 13.86 (10.37) and 18.98 (12.44), respectively (Table 2). The distributions of ΔCDI-S and ΔSCARED were shown in Supplementary Figure S1. The mean score of CDI-S and SCARED for the two waves was reported in Supplementary Figure S2A. For both scales, we found that the score was increased at Wave 2 compared with those at Wave 1 for each grade. The mean and standard deviations of ΔCDI-S and ΔSCARED were 1.35 (3.68) and 5.09 (14.31), respectively (Table 2). As shown in Supplementary Figure S2B, children in Wuhan had a higher change of SCARED score than those in Huangshi.

**Table 2:** Distribution of scale scores of children who completed both surveys.

		**CDI-S**	**SCARED**
Wave 1			
	Symptoms, No. (%)	250 (20.4)	232 (19.0)
	No symptoms, No. (%)	974 (79.6)	992 (81.0)
	Mean and standard deviation	2.22 (2.49)	13.86 (10.37)
Wave 2			
	Symptoms, No. (%)	487 (39.8)	406 (33.2)
	No symptoms, No. (%)	723 (59.1)	811 (66.3)
	Mean and standard deviation	3.57 (3.29)	18.98 (12.44)
	Missing data, No. (%)	14 (1.1)	7 (0.6)
Δ score			
	Mean and standard deviation	1.35 (3.68)	5.09 (14.31)
	1st tertile, No. (%)	539 (44.0)	414 (33.8)
	Δ score range	<1	<0
	2nd tertile, No. (%)	308 (25.2)	419 (34.2)
	Δ score range	≥ 1, <3	≥ 0, <11
	3rd tertile, No. (%)	363 (29.7)	384 (31.4)
	Δ score range	≥ 3	≥ 11
	Missing data, No. (%)	14 (1.1)	7 (0.6)

*Δ score was change of scale scores from Wave 1 to Wave 2*.

*CDI-S, Children's Depression Inventory-Short Form; SCARED, The Screen for Child Anxiety Related Emotional Disorders*.

As shown in Table 3, the prevalence of mental health outcomes among children at Wave 2 significantly increased from those levels at Wave 1, specifically in depressive symptoms [39.8% (Wave 2) vs. 20.4% (Wave 1), *p* < 0.001] and anxiety symptoms [33.2% (Wave 2) vs. 19.0% (Wave 1), *p* < 0.001]. Further subset analyses for gender, grade, and school location showed similar results (all *p* < 0.001). Tables 4, 5 showed the OR and β for associations of Δ score and demographic characteristics in the regression models. Children with experience of neglect had higher ΔCDI-S scores (β = 1.12; 95% CI = 0.67–1.58) and ΔSCARED score (β = 6.46; 95% CI = 4.73–8.19) compared with those without neglect. Children with experience of neglect had higher odds in the 3rd tertile of the ΔCDI-S score (OR = 2.51; 95%CI = 1.82–3.47). Similar results were found for ΔSCARED score (2nd tertile vs. 1st tertile, OR = 1.37; 95% CI = 1.02–1.84; 3rd tertile vs. 1st tertile, OR = 3.46; 95% CI = 2.45–4.89). Females had significantly higher Δ score of SCARED than males (β = 1.83; 95% CI = 0.26–3.40) and children in Wuhan had significantly higher ΔSCARED score than those in Huangshi (β = 3.42; 95% CI = 1.77–5.07). Children in Wuhan had higher odds in the third tertile of ΔCDI-S score (OR = 1.38; 95% CI = 1.03–1.83) and the third tertile of ΔSCARED score (OR = 1.65; 95% CI = 1.22–2.25). We also found that students in Grade 4 and 5 had lower ΔCDI-S score compared with those students in Grade 3 (β = −0.93; 95% CI = −1.50 to −0.36; β = −0.68; 95% CI = −1.23 to −0.14). The sensitivity analyses that used complete data before multiple imputation showed similar results (Supplementary Tables S1, S2).

**Table 3:** Change of psychological symptoms outcomes among children at two surveys.

**Characteristics**	**Depressive symptoms**			**Anxiety symptoms**		
	**Wave 1 Yes, n (%)**	**Wave 2 Yes, n (%)**	***P*-value**	**Wave 1 Yes, n (%)**	**Wave 2 Yes, n (%)**	***P*-value**
Overall	250 (20.4)	487 (39.8)	<0.001	232 (19.0)	406 (33.2)	<0.001
**Gender**						
Male	145 (21.0)	276 (40.6)	<0.001	126 (18.3)	199 (29.1)	<0.001
Female	105 (19.6)	211 (39.8)	<0.001	106 (19.8)	207 (38.8)	<0.001
**School location**						
Wuhan	106 (25.3)	193 (46.2)	<0.001	79 (18.9)	176 (42.0)	<0.001
Huangshi	144 (17.9)	294 (37.1)	<0.001	153 (19.0)	230 (28.8)	<0.001
**Grade**						
Grade 3	48 (14.2)	124 (37.5)	<0.001	54 (16.0)	106 (31.8)	<0.001
Grade 4	63 (21.6)	107 (37.2)	<0.001	56 (19.2)	112 (38.6)	<0.001
Grade 5	80 (23.5)	145 (42.9)	<0.001	78 (22.9)	107 (31.5)	0.006
Grade 6	59 (23.1)	111 (43.9)	<0.001	44 (17.3)	81 (31.9)	<0.001

*P-value was derived from McNemar's test*.

*Depressive symptoms were measured by the Children's Depression Inventory-Short Form*.

*Anxiety symptoms were measured by the Screen for Child Anxiety Related Emotional Disorders*.

**Table 4:** Association between demographic characteristics and the difference in Children's Depression Inventory-short form (ΔCDI-S) score.

**Characteristic**	**2nd tertile (≥1, <3)**	**3rd tertile (≥3)**	**Linear model**
	**OR (95%CI)**	**OR (95%CI)**	**β (95%CI)**
**Gender**			
Female vs. Male	0.81 (0.61,1.07)	0.94 (0.72,1.24)	0.20 (−0.21,0.61)
School location			
Wuhan vs. Huangshi	1.35 (1.00,1.82)	1.38 (1.03,1.83)	0.33 (−0.10,0.76)
Grade			
Grade 4 vs. 3	0.80 (0.54,1.19)	0.72 (0.49,1.05)	−0.93 (−1.50, −0.36)
Grade 5 vs. 3	1.02 (0.71,1.45)	0.77 (0.54,1.12)	−0.68 (−1.23,- –0.14)
Grade 6 vs. 3	0.94 (0.62,1.43)	1.07 (0.72,1.58)	−0.20 (−0.80,0.39)
**Protective measures during COVID-19 (Wave 1)**			
Yes vs. No	0.95 (0.71,1.26)	0.91 (0.69,1.19)	0.12 (−0.29,0.53)
**Knowing the host of SARS-CoV-2 (Wave 1)**			
Yes vs. No	0.90 (0.66,1.22)	1.15 (0.85,1.56)	0.32 (−0.13,0.77)
**Daily sleep time (Wave 2)**			
<8 vs. ≥ 8 h	1.12 (0.83,1.53)	1.19 (0.85,1.67)	0.29 (−0.20,0.78)
**Neglect (Wave 2)**			
Yes vs. No	1.34 (0.99,1.83)	2.51 (1.82,3.47)	1.12 (0.67,1.58)

*Ref, Reference; OR, odds ratios; CI, confidence intervals; CDI-S, Children's Depression Inventory-Short Form*.

*ΔCDI-S score was the change of scale scores from Wave 1 to Wave 2*.

*OR (95% CI) were derived from the multinomial logistic regression model and the first tertile was the reference group (score < 1)*.

*β (95% CI) were derived from generalized linear regression*.

**Table 5:** Association between demographic characteristics and difference in Screen for Child Anxiety Related Emotional Disorders (ΔSCARED) score.

**Characteristics**	**2nd tertile (≥0, <11)**	**3rd tertile (≥11)**	**Linear model**
	**OR (95%CI)**	**OR (95%CI)**	**β (95%CI)**
**Gender**			
Female vs. Male	1.03 (0.78,1.36)	1.30 (0.98,1.74)	1.83 (0.26,3.40)
School location			
Wuhan vs. Huangshi	1.47 (1.09,1.98)	1.65 (1.22,2.25)	3.42 (1.77,5.07)
**Grade**			
Grade 4 vs. 3	1.04 (0.70,1.54)	1.03 (0.69,1.54)	0.63 (−1.55,2.80)
Grade 5 vs. 3	1.02 (0.73,1.44)	0.71 (0.48,1.04)	−1.58 (−3.67,0.50)
Grade 6 vs. 3	1.01 (0.68,1.51)	0.91 (0.60,1.39)	−0.24 (−2.52,2.03)
**Protective measures during COVID-19 (Wave 1)**			
Yes vs. No	0.94 (0.71,1.23)	0.88 (0.66,1.17)	−0.51 (−2.08,1.06)
**Knowing the host of SARS-CoV-2 (Wave 1)**			
Yes vs. No	0.88 (0.65,1.19)	0.98 (0.71,1.35)	0.55 (−1.17,2.27)
**Daily sleep time (Wave 2)**			
<8 vs. ≥ 8 h	1.25 (0.92,1.70)	1.27 (0.92,1.77)	0.62 (−1.14,2.37)
**Neglect (Wave 2)**			
Yes vs. No	1.37 (1.02,1.84)	3.46 (2.45,4.89)	6.46 (4.73,8.19)

*Ref, Reference; OR, odds ratios; CI, confidence intervals; SCARED, Screen for Child Anxiety Related Emotional Disorders*.

*ΔSCARED score was the change of scale scores from Wave 1 to Wave 2*.

*OR (95% CI) were derived from the multinomial logistic regression model and the first tertile was the reference group (score < 0)*.

*β (95% CI) were derived from the generalized linear regression*.

## Discussion

This study suggested that about 3 months after school reopening, the prevalence of depressive and anxiety symptoms among children in Hubei province remained elevated compared with that during the COVID-19 pandemic lockdown. When considering the ΔCDI-S and ΔSCARED scores, the risk factors for a high change from Wave 1 to Wave 2 were: the school in Wuhan, being female, and having experience of neglect.

The psychological and mental effects of major public health events could be long term (38–40). Lessons from the outbreak of severe acute respiratory syndrome (SARS) in 2003 indicated that the mental health of survivors did not improve over time and gradually deteriorated (41). The post-traumatic disturbance of residents in areas with high SARS prevalence, regardless of age, was more intense than in areas with low prevalence (42). A national mental health study among adolescents in China, administered separately in February and April 2020, showed that the prevalence of depression and anxiety significantly increased over time (43). In addition, surveys covering 5,285 adults in the USA found that the prevalence of adverse mental health symptoms during the later phase of the COVID-19 pandemic (September 2020) was higher than in June 2020 (44). Daly et al. found that a pronounced and prolonged deterioration of mental health occurred between April and June 2020 among participants of the nationally representative United Kingdom Household Longitudinal Study (45). Studies in Italy showed an increase in stress and depression among citizens along with a different time course of mental health problems between men and women (46, 47). Our results among children in Hubei province, China, were consistent with these findings. Although different socio-cultural contexts (i.e., tight and loose cultures) led to a varied response to a global pandemic (48), COVID-19 seemed to have a similar impact on the long-term consequences of mental health.

The significant increase in the prevalence of depressive and anxiety symptoms may be related to the fact that an online mental health service in the early phase of COVID-19 in China was not designed for children (49). Children who developed psychological symptoms at Wave 1 may persist with these symptoms until Wave 2 due to lack of effective intervention. For children with depressive symptoms, there will be considerable difficulties in resuming normal life after school reopening (50). The other important thing to note in this study was that we used screening criteria, rather than clinical thresholds, of the CDI-S (≥ 7) (51) and SCARED (≥ 25) (52). This was because we tried to screen out more children at high or potential risk from the aspect of early prevention, especially for the children in Wuhan who experienced the pandemic earlier and more severely. Although the sample size was limited and is not fully representative of the population in Hubei province, the evidence of increased depressive and anxiety symptoms suggested that there is a great need to provide timely psychological support to enhance resilience and reduce fear and anxiety (53). On a related note, timely mental health education and treatment should be available for these children (54).

Consistent with previous findings, females had higher SCARED scores in our study (43, 55). The gender difference in anxiety symptoms may be partly attributable to relationships between adrenarcheal hormones and functional connectivity of the amygdala according to an imaging study in children (56). Hormone levels in females were inversely associated with the connection from the right amygdala to the insula, but were positively associated with the connection from the left amygdala to anterior cingulate cortex in males. Furthermore, we found that children in Wuhan at Wave 2 had a higher ΔSCARED score than those in Huangshi, which may be attributed to the fact that the epidemic in Wuhan was more severe than in Huangshi, and that children in Wuhan have been isolated at home for longer periods (57). Moreover, we found higher ΔCDI-S and ΔSCARED scores at Wave 2 in children with experience of neglect in the preceding year vs. those without neglect. The experience of neglect over the past year also included the children's experience at the time of the first survey. This may be partly attributed to the fact that children might have a decreased frequency of positive parent–child interaction after the school reopened, which increased the probability of neglect (58). Changes to daily family life due to financial hardship and social restrictions on parents may increase parental stress and lead to an increase in adverse childhood experiences (ACEs), including neglect (59). In this study, 69.8% of children reported experience of neglect in the preceding year at Wave 2, which was higher than a previous study among Chinese elementary students in Shanghai, China (52.26%) (60). ACEs, such as abuse and neglect, are associated with increased risk for depression, anxiety, and PTSD (61), along with elevated mortality rates (62). Although Chinese parents have a more democratic parenting style influenced by Western thoughts, the power disparity between parents and children in traditional Chinese culture may facilitate ACEs. Support for dealing with family difficulties and available child welfare services are needed.

Although we explored psychological problems among the cohort of children in Hubei province, China, there were several limitations. First, the results may be generalized only to children in school. We adopted a cluster sampling method and selected two primary schools for the surveys. The sample was therefore not necessarily representative of the whole population of children in China. Second, no information on household income or other types of ACEs was surveyed. The impact of the COVID-19 pandemic may be related to parental unemployment/loss of household income and high-stress home environments, thus increasing the likelihood of ACEs or emotional problems (63). We also did not collect information related to family functioning or family context. Third, children in higher grades were more likely to suffer from mental health problems (64). The students in Grade 6 at Wave 1 were lost during follow-up due to promotion from primary school to junior middle school, which may lead to an underestimation of the prevalence of depressive and anxiety problems at Wave 2. Fourth, we did not collect information on learning styles. The learning styles were inconsistent between the two surveys (home learning vs. studying on campus), which may have an impact on the mental health of students. Furthermore, we used electronic questionnaires when students were confined to home and paper-based questionnaires when students were at school. Although we adopted some methods to ensure that students completed the questionnaires independently, we still need to unify the form of survey tools in future studies. Finally, we reported the symptoms rather than the clinical diagnoses because of the short follow-up period.

In conclusion, our study identified increased prevalence of depressive and anxiety symptoms among a cohort of children in Hubei province, China, despite the fact that the COVID-19 pandemic had been brought under control and schools had reopened. The mental health problems of children are warnings. There is a lack of knowledge on the long-term psychological impact of COVID-19 on children, and our results fill an important gap in the research. In addition, China is one of the early affected countries whose schools are now functioning normally. Our study, focusing on the progression of psychological symptoms in children who have experienced long-term home quarantine and have now resume school, may guide the mental health support plan in other countries (65). We anticipate that our results may be helpful to decision makers and that post-COVID-19 public health for mental health protection be given priority. Schools, which are the primary provider of mental health services for many children (66), should take timely action to mitigate the disruption of COVID-19 on children when they return to school, especially those who have experienced neglect within their families (2). For psychiatrists and healthcare professionals, they may participate in educational and media activities for children, parents, or educators about the mental health distress caused by physical distancing and quarantine. They should also alert policy makers of the long-term consequences of COVID-19 and the increased demand for mental health services (67). Continuing to follow-up these children and giving attention to their emotional problems is also necessary.

## Data Availability Statement

The datasets presented in this article are not readily available because the data are not publicly available due to privacy/ethical restrictions: Requests to access the datasets should be directed to RS, songranran@hust.edu.cn.

## Ethics Statement

The studies involving human participants were reviewed and approved by the Ethics Committee of Tongji Medical College, Huazhong University of Science and Technology. Written/oral informed consent to participate in this study was provided by the participants' legal guardian/next of kin.

## Author Contributions

XX and RS conceived the study. XX, QL, KZ, YF, and RS critically appraised the data. XX, KZ, QJ, XW, and PX prepared the initial manuscript. RS reviewed and edited the manuscript. All authors collected data for the study and critically reviewed and approved the final manuscript.

## Conflict of Interest

The authors declare that the research was conducted in the absence of any commercial or financial relationships that could be construed as a potential conflict of interest.

## Publisher's Note

All claims expressed in this article are solely those of the authors and do not necessarily represent those of their affiliated organizations, or those of the publisher, the editors and the reviewers. Any product that may be evaluated in this article, or claim that may be made by its manufacturer, is not guaranteed or endorsed by the publisher.
