# Supplementary material for: Psychological Symptom Progression in School-Aged Children After COVID-19 Home Confinement: A Longitudinal Study
Source: Front Psychiatry. 2022 Apr 13;13:809107. doi: 10.3389/fpsyt.2022.809107 (PMC9043353; doi:10.3389/fpsyt.2022.809107)
Supplement: Supplementary file 1 [file Data_Sheet_1.docx]

Supplementary Material

R code for Figure S2

Table S1 Sensitivity analyses of association between demographic characteristics and ΔCDI-S score using complete data.

Table S2 Sensitivity analyses of association between demographic characteristics and ΔSCARED score using complete data.

Figure S1 The distribution of ΔCDI-S and ΔSCARED score.

Figure S2 CDI-S and SCARED score distribution in elementary students. (A) Distribution of CDI-S and SCARED score at two waves in Grades 3–6. The vertical line in each distribution represents the mean. Blue represents March and red represents November. (B) Distribution of ΔCDI-S and ΔSCARED score in Wuhan and Huangshi and in Grades 3–6. The vertical line in each distribution represents the mean. Blue represents Wuhan and red represents Huangshi.

R code for Figure S2

p <- ggplot(data = data, mapping = aes(

x = score,

y = Time,

fill = Time))

p + geom_density_ridges(alpha = 0.5,

quantile_lines=TRUE,

quantile_fun=function(x,...)mean(x)) +

guides(fill = FALSE) +

labs(x = "Scale score",

y = "Time")+facet_grid(grade~group,scales="free_x")+

theme(axis.title.x=element_text(size=30),

axis.title.y=element_text(size=30),

axis.text.x=element_text(size=30),

axis.text.y=element_text(size=30),

strip.text = element_text( size = 30),

text=element_text(family="Arial"),

panel.grid=element_blank()

)

p <- ggplot(data = data1, mapping = aes(

x = score,

y = location,

fill = location))

p + geom_density_ridges(alpha = 0.5,

quantile_lines=TRUE,

quantile_fun=function(x,...)mean(x)) +

guides(fill = FALSE) +

labs(x = "Δ Score",

y = "Location")+facet_grid(grade~group,scales="free_x")+

theme(axis.title.x=element_text(size=30),

axis.title.y=element_text(size=30),

axis.text.x=element_text(size=30),

axis.text.y=element_text(size=30),

strip.text = element_text( size = 30),

panel.grid=element_blank()

)

Table S1 Sensitivity analyses of association between demographic characteristics and ΔCDI-S score using complete data.

| Characteristics | | 2nd tertile (≥1, < 3) | 3rd tertile (≥ 3) | Linear model |
| --- | --- | --- | --- | --- |
|  |  | OR (95%CI) | OR (95%CI) | β (95%CI) |
| Gender | |  |  |  |
|  | Female vs. Male | 0.81 (0.59,1.11) | 0.87 (0.64,1.19) | 0.01 (-0.44,0.46) |
| School location | |  |  |  |
|  | Wuhan vs. Huangshi | 1.52 (1.05,2.19) | 1.14 (0.78,1.67) | 0.06 (-0.48,0.60) |
| Grade | |  |  |  |
|  | Grade 4 vs. 3 | 0.87 (0.56,1.34) | 0.80 (0.52,1.23) | -0.69 (-1.31,-0.07) |
|  | Grade 5 vs. 3 | 1.05 (0.70,1.59) | 0.85 (0.56,1.29) | -0.59 (-1.19,0.01) |
|  | Grade 6 vs. 3 | 0.91 (0.56,1.46) | 1.10 (0.70,1.73) | -0.04 (-0.71,0.63) |
| Protective measures during COVID-19 (Wave 1) | | |  |  |
|  | Yes vs. No | 0.90 (0.65,1.23) | 1.02 (0.75,1.40) | 0.19 (-0.26,0.65) |
| Knowing the host of SARS-CoV-2 (Wave 1) | | |  |  |
|  | Yes vs. No | 0.85 (0.61,1.20) | 1.03 (0.73,1.46) | 0.05 (-0.45,0.54) |
| Daily sleep time (Wave 2) | |  |  |  |
|  | < 8 hours vs. ≥ 8 hours | 1.21 (0.86,1.70) | 1.22 (0.87,1.71) | 0.34 (-0.15,0.82) |
| Neglect (Wave 2) | |  |  |  |
|  | Yes vs. No | 1.35 (0.96,1.89) | 2.77 (1.91,4.00) | 1.14 (0.66,1.63) |

Ref: Reference; OR: odds ratios; CI: confidence intervals; CDI-S: Children’s Depression Inventory-Short Form

ΔCDI-S score was the change of scale scores from Wave 1 to Wave 2.

OR (95% CI) were derived from the multinomial logistic regression model and the first tertile was the reference group (score < 1).

β (95% CI) were derived from generalized linear regression.

Table S2 Sensitivity analyses of association between demographic characteristics and ΔSCARED score using complete data.

| Characteristics | | 2nd tertile (≥0, < 11) | 3rd tertile (≥ 11) | Linear model |
| --- | --- | --- | --- | --- |
|  |  | OR (95%CI) | OR (95%CI) | β (95%CI) |
| Gender | |  |  |  |
|  | Female vs. Male | 1.05 (0.77,1.42) | 1.26 (0.91,1.74) | 1.58 (-0.19,3.34) |
| School location | |  |  |  |
|  | Wuhan vs. Huangshi | 1.51 (1.04,2.21) | 1.69 (1.14,2.51) | 4.20 (2.09,6.32) |
| Grade | |  |  |  |
|  | Grade 4 vs. 3 | 0.99 (0.65,1.53) | 1.16 (0.75,1.81) | 1.29 (-1.15,3.73) |
|  | Grade 5 vs. 3 | 1.09 (0.73,1.62) | 0.79 (0.51,1.22) | -0.87 (-3.21,1.48) |
|  | Grade 6 vs. 3 | 1.12 (0.71,1.76) | 1.11 (0.68,1.80) | 0.87 (-1.77,3.50) |
| Protective measures during COVID-19 (Wave 1) | | |  |  |
|  | Yes vs. No | 0.96 (0.71,1.31) | 0.91 (0.66,1.26) | -0.28 (-2.05,1.50) |
| Knowing the host of SARS-CoV-2(Wave 1) | | |  |  |
|  | Yes vs. No | 0.90 (0.64,1.26) | 0.96 (0.67,1.38) | -0.04 (-1.99,1.91) |
| Daily sleep time (Wave 2) | |  |  |  |
|  | < 8 hours vs. ≥ 8 hours | 1.29 (0.93,1.80) | 1.29 (0.91,1.83) | 0.97 (-0.94,2.87) |
| Neglect (Wave 2) | |  |  |  |
|  | Yes vs. No | 1.42 (1.03,1.96) | 3.51 (2.39,5.16) | 6.29 (4.37,8.21) |

Ref: Reference; OR: odds ratios; CI: confidence intervals; SCARED: Screen for Child Anxiety Related Emotional Disorders.

ΔSCARED score was the change of scale scores from Wave 1 to Wave 2.

OR (95% CI) were derived from the multinomial logistic regression model and the first tertile was the reference group (score < 0).

β (95% CI) were derived from the generalized linear regression.


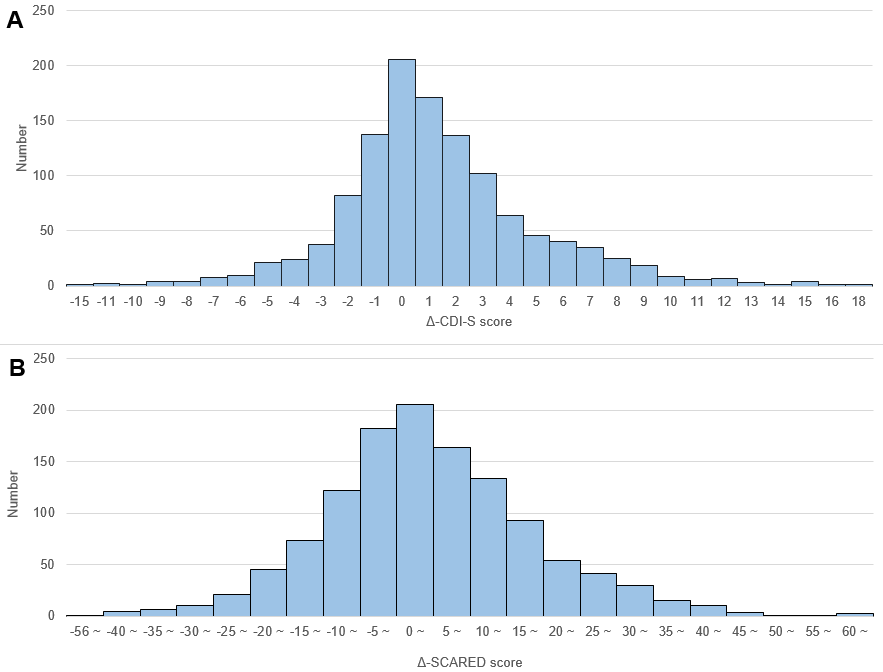


Figure S1 The distribution of ΔCDI-S and ΔSCARED score.


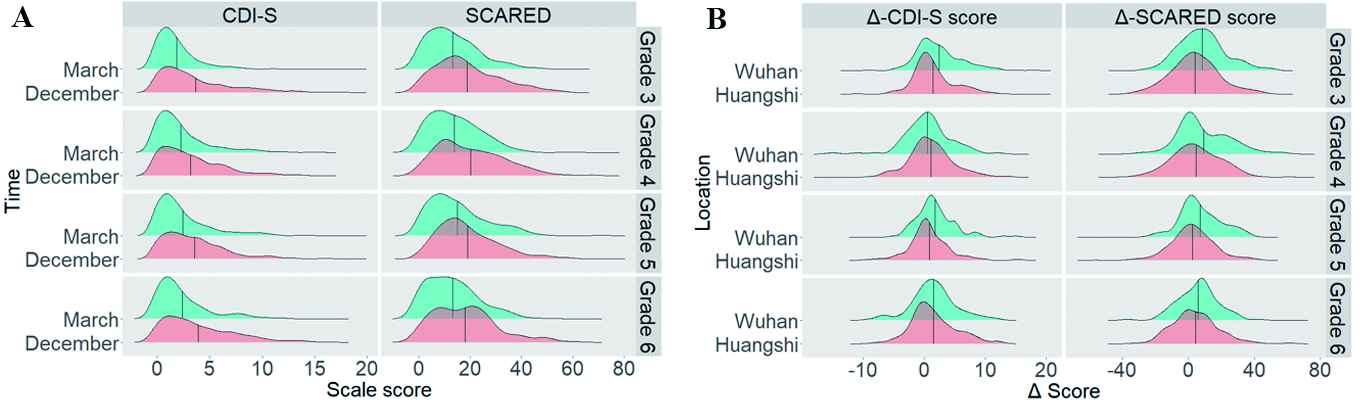
Figure S2 CDI-S and SCARED score distribution in elementary students. (A) Distribution of CDI-S and SCARED score at two waves in Grades 3–6. The vertical line in each distribution represents the mean. Blue represents March and red represents November. (B) Distribution of ΔCDI-S and ΔSCARED score in Wuhan and Huangshi and in Grades 3–6. The vertical line in each distribution represents the mean. Blue represents Wuhan and red represents Huangshi.
